# Chronic Cluster Headache with an Atypical Presentation and Treatment Response

**DOI:** 10.1155/2016/5230127

**Published:** 2016-12-29

**Authors:** Telma Santos, Hugo Morais

**Affiliations:** Neurology Department, Centro Hospitalar Vila Nova de Gaia/Espinho, Porto, Portugal

## Abstract

The management of cluster headache (CH) may be challenging. We report a 50-year-old male with recurrent attacks of dull and severe unilateral periorbital pain, lasting 30–45 minutes, twice a day, exclusively during sleep, and accompanied by ipsilateral rhinorrhea and lacrimation. The pain switched sides within every attack. CH treatment was initiated but the patient maintained recurrence rates compatible with chronic CH, even after increasing verapamil to 460 mg/day. Afterwards we decided to add lithium (800 mg/day). With this treatment the severity and recurrence of CH substantially decreased, despite the patient's autonomous decision to take lithium only during the acute phase of the cluster. The exclusively alternating location and the excellent response to short cycles of lithium represent two unique features of CH.

## 1. Introduction

Cluster headache (CH) comprises the most frequent trigeminal-autonomic headache syndrome and has a mean prevalence of 0,1% [1]. The designation highlights its typical pattern, in which each attack (a single episode of headache) occurs in clusters or bouts of variable duration, with a circadian and circannual rhythmicity [1, 2].

The latest version of the International Classification of Headache Disorders (ICHD3b) defines CH in the setting of at least five attacks of severe unilateral headache, in the orbital, supraorbital, and/or temporal regions, each lasting 15 to 180 minutes (when untreated) and occurring one every other day to eight per day [3]. Additionally, a sense of restlessness (or agitation) and/or at least one symptom/sign of autonomic dysfunction (conjunctival injection, lacrimation, nasal congestion, rhinorrhea, eyelid oedema, sweating, flushing, sensation of ear fullness, miosis, and ptosis), ipsilateral to the headache, must be present [3]. CH may also present atypical features, such as a distinct frequency or duration of the attacks and the association with atypical symptoms or signs [4].

Regarding the location of the pain, although the majority of patients report a lateralized headache, 15% may refer to an alternating pattern. This side-switching is mostly verified between clusters, is extremely rare within a single bout, and never occurs within the same attack [1, 2].

CH is classified into episodic (Episodic CH, ECH) or chronic (Chronic CH, CCH) forms according to headache frequency [1–3]. The most prevalent is ECH, which is characterized by bouts lasting more than 1 week separated by remissions lasting longer than 4 weeks [1–3]. CCH is defined by the absence of remissions within one year, or with remission periods lasting less than 1 month [1–3]. It is estimated that 10–20% of patients develop CCH, usually from a preexisting ECH, with only 5% presenting a CCH from the onset [1, 2].

The pharmacological management of CH is challenging and requires the combination of three strategies: acute treatment, to abort a single attack; transitional treatment, to stop the cluster; and prophylactic treatment to prevent further recurrences [5–7].

Despite sharing the same treatment strategies with ECH, CCH may be more difficult to control, which demand an efficient prophylactic strategy [5]. CCH patients also require an even closer clinical follow-up as they are exposed to a higher risk of drug's side-effects due to their prolonged use [5].

Lithium is used in prophylactic treatment of CH, as an alternative to verapamil (when contraindicated) or in an add-on scheme [5–7].

The authors report a unique case of a CCH that alternate sides within the same cluster and between attacks, demonstrating a significant response to short cycles of lithium.

## 2. Case Report

A 50-year-old Caucasian male was referred to our outpatient clinic because of recurrent attacks of periorbital pain. The patient described a dull pain located in the periorbital region with an intensity of 10/10 in Numeric Pain Rating Scale. Each attack lasted between 30 and 45 minutes and occurred twice a day, every day for the last three months, exclusively during sleep, and always between 22.30 and 23.30 h and 0.30 and 1.15 h. The patient emphasizes that the pain switched sides between one attack and another but not during the same attack. The pain was accompanied by rhinorrhea and lacrimation whose location always respected the location of the pain (the autonomic signs were always ipsilateral to the site of the pain). He also refers to feeling agitated during each attack. He denied other symptoms/signs of autonomic dysfunction, nausea, vomiting, and light, sound, or movement intolerance. He also denied any alleviating or precipitant factor.

The patient had medical history of dyslipidemia, treated with 20 mg simvastatin. He denied smoking habits. The personal and family medical history was unremarkable for migraine or other headache disorders.

The neurological examination, including fundoscopy, was normal.

The diagnosis of a CH was established and the patient was informed about the disease and treatment options. Oxygen (12 L/min, portable home device) and 5 mg intranasal zolmitriptan were prescribed for acute treatment. A 6-day course of 60 mg prednisone and 120 mg bid verapamil (he had a normal EKG) were also prescribed. We decided not to perform an indomethacin test and we have not tried a preventive treatment with caffeine. A brain-MRI was asked and the patient was advised to return in two weeks.

However, he returned after ten months referring to a partial improvement. After starting treatment the attacks became less severe, occurring once a day (between 22.30 and 23.30 h) in clusters of 2–4 days with a frequency of 2-3 per month. The acute therapy (intranasal zolmitriptan and oxygen) ceased the attacks in about 10–15 minutes. The brain-MRI was normal. Because of loss of visual acuity, he recently visited an ophthalmologist who diagnosed bilateral corticogenic cataracts. He was then advised to stop corticosteroids and to increase verapamil in a stepwise manner to 240 mg bid (with ECG monitorization). Due to the lack of benefit after three months, we decided to introduce 400 mg bid lithium as an add-on to verapamil.

The patient noticed a significant effect just after lithium introduction. He started this treatment in the first day of the next cluster and noticed a significant decrease in severity and duration. Consequently, he decided to stop lithium and maintain verapamil against the dosage regimen. Remarkably, the following cluster only recurred after one month, in which the patient added lithium again, ceasing that cluster in 2 days. The patient reported a decrease of more than 80% on the number of days with headache per month, with a substantial improvement in his quality of life. So, he decided to maintain his strategy: 12 L oxygen, 5 mg intranasal zolmitriptan, and 400 mg bid lithium during the acute phase and 240 mg bid verapamil as a prophylactic. In the last visit he reported only two clusters lasting three days in the previous six months.

## 3. Discussion

CH may represent a diagnostic and therapeutic challenge.

The authors report a CCH that combine some unique features.

First, the patient insists that his periorbital pain always switches sides from one attack to another. In a review that gathered the clinical manifestations of 180 CH patients, 28 (15%) presented a side-switching headache, 9 of which (5%) alternates within the same cluster [8]. However, to the author's best knowledge, an exclusively alternating pattern between CH attacks has not been described elsewhere. The pathophysiology of CH lies in the activation of trigeminal-autonomic reflex. However, the anatomical and pathophysiological correlates of CH do not offer a clear explanation for the exclusive side-switching presented by our patient.

Moreover, our patient manifested another atypical clinical feature: the attacks manifests exclusively during sleep.

As the attacks developed exclusively during sleep and showed an excellent response to lithium, hypnic headache (HH) comprises the most important differential diagnosis. HH typically affects middle-aged adults (mean age of 62 years), has a dull character and a moderate intensity, and is localized in frontotemporal regions (including periorbital) in the majority of patients [9, 10]. Untreated, the attacks last about 1.5 hours (from 15 to 600 minutes) and have a mean frequency of one every 24 hours (from one per week to six per night) [9, 10]. According to the ICHD3, the HH does not have autonomic symptoms/signs [3]. Curiously, a few HH patients have been described to present autonomic features (lacrimation, rhinorrhea, and ptosis) but did not fulfill clinical criteria for CH or CPH [9, 10]. Lithium is the drug of choice in the treatment of HH [9, 10]. The pathophysiology of HH is unclear but has been debated whether hypnic headache is a particular subtype of cluster headache [9, 10]. In our case, the exclusive occurrence of pain during sleep time and the excellent response to lithium may favor a possible common pathophysiology between both disorders.

Chronic Paroxysmal Hemicrania (CPH) comprises another differential diagnosis in this case. CPH is also a trigeminal-autonomic headache that differs from the CCH in the attack duration (2–30 minutes), attack frequency (1–45 minutes), and response to indomethacin [11]. An oral indomethacin trial or Indotest (intramuscular injection 100 mg indomethacin) are extremely useful to distinguish CPH from CCH in cases with overlapping features and refractory to the usual treatments of cluster headache [12]. Intramuscular indomethacin is not available in our country. In our patient, all the attacks lasted more than 30 minutes, had a daily frequency of only 1 or 2 attacks, and had a notorious effect after acute treatment of cluster headache (oxygen and zolmitriptan), which support the diagnosis of a cluster headache. So, we decided not to perform an oral indomethacin trial.

The other unique feature of our case relates the response to the treatment. The general treatment of CH is summarized in Table 1 [5–7].

CCH demands an aggressive treatment focused on an effective prophylaxis. The nondihydropyridine calcium channel blocker verapamil in an adequate dosage is regarded as the basis of CCH prophylaxis [5–7]. Adverse effects are usually dose-dependent and include constipation, dizziness, hypotension, oedema, hypotension, and bradycardia, due to his antiarrhythmic, antianginal, vessel dilating, and negative inotropic actions [5–7]. Consequently, ECG monitoring is recommended for each 80 mg increase to a daily dosage equal or superior to 480 mg [5–7].

Lithium is the first alternative treatment to verapamil [5–7]. This drug has a narrow therapeutic window and lithium serum levels should be monitored and kept between 0.6 mmol/L and 1.2 mmol/L [5–7]. Liver, renal, and thyroid functions and electrolytes should also be monitored regularly to prevent adverse effects [5–7].

The mechanism of action of lithium is still unclear. It is known to interfere with multiple neuronal pathways involving glutamate, serotonin, glycogen synthase kinase-3, and inositol that explain his antidepressant and mood-stabilizing proprieties, but not its effect in CH [13]. Lithium has the ability to act either at a physiological level on the period, phase, amplitude, and coupling of biological rhythms or at a molecular level on circadian gene expression and protein production [14]. Moreover, this drug seems to interfere in the retinal hypothalamic pineal pathway to interact with environmental light and influence circadian rhythms [14]. We speculate that its action on sleep patterns and in circadian rhythms may be responsible for the significant benefit in our patient.

This patient presented a CCH with a poor response to adequate dosages of verapamil, but obtained a substantial decrease in cluster severity, duration, and frequency after starting lithium as an add-on. In fact, a review of open clinical trials found a response rate of 78% in CCH, compared with 63% obtained in ECH group [15]. Moreover, a double-bind controlled trial found better results for the group on lithium (800 mg/day), compared with placebo (62% versus 43%) [16].

Remarkably, our patient noticed that by taking lithium exclusively in the acute phase (in addition to oxygen and triptan), against the prescribed prophylactic regimen, the cluster would become milder, shorter, and less prone to recur. This unusual finding may be explained by an optimal response to the drug. In fact, despite the fact that most patients report a benefit only after several weeks of treatment, a response may be recognized in the first week [5–7]. Furthermore, the efficacy of lithium in CCH may be durable up to 4 years after drug suspension and may evolve to an ECH [15], as we observed in our patient. However, despite being a consistent feature, it is obviously possible that the rare bouts of CH after lithium introduction may rather stop spontaneously and not due to drug therapy.

Finally, we would like to emphasize the erroneous behavior of our patient on avoiding medical advices and adjusting his own medication without medical support. In fact, in studies of psychological profile, paranoid-schizoid personality traits and mood disorders are more frequent among CH patients that might influence their response to therapy and their therapeutic alliance with neurologists [17–19]. In such cases, a proper follow-up might prove to be more difficult to ensure because of lack of patient adherence.

In summary, the exclusively alternating location and the excellent response to short cycles of lithium comprise two unusual features of CH.

Albeit rare, an exclusively alternating pain may be a manifestation of CH. Furthermore, an excellent response of lithium used only during the acute phase is unusual. This may obviously be just an anecdotal finding. However, if future randomized clinical trials eventually suggest a possible benefit, this strategy would have the advantage of preventing side-effects due to the prolonged use of prophylactic drugs.

## Figures and Tables

**Table 1:** Summary of treatment options in CH [4–6].

	Medications	Level of evidence	Line	Dosage
*Acute treatment*	Oxygen	A	First line	100% oxygen 12/15 L/min
	Sumatriptan sc	A	First line	6 mg
	Sumatriptan in	B	First line	20 mg
	Zolmitriptan in	A	First line	5–10 mg

*Transitional treatment*	Prednisone	C	First line	60–100 mg
	Occipital nerve block	B	First line	Methylprednisolone slow-release, cortivazol (3.75 mg in 1.5 mL saline), plus lidocaine
	Dihydroergotamine	C	Alternative	1 mg sc/im

*Prophylactic treatment*	Verapamil	B	First line	360 mg/day (240–960)
	Lithium	C	First line	900 mg/day (600–1200)
	Valproic acid	C	Second line	500–2000 mg/day
	Topiramate	B	Second line	50–200 mg/day
	Baclofen	C	Second line	15–30 mg/day
	Melatonin	C	Alternative	10 mg/day

sc: subcutaneous; im: intramuscular; level of evidence: A, data derived from multiple randomized clinical trials or meta-analyses; B, data derived from a single randomized clinical trial or large nonrandomized studies; C, consensus of opinion of the experts and/or small studies, retrospective studies, and registries.
